# A machine learning algorithm-based risk prediction score for in-hospital/30-day mortality after adult cardiac surgery

**DOI:** 10.1093/ejcts/ezae368

**Published:** 2024-10-07

**Authors:** Shubhra Sinha, Tim Dong, Arnaldo Dimagli, Andrew Judge, Gianni D Angelini

**Affiliations:** Department of Cardiac Surgery, Bristol Heart Institute, Translational Health Sciences, University of Bristol, UK; Department of Cardiac Surgery, Bristol Heart Institute, Translational Health Sciences, University of Bristol, UK; Department of Cardiac Surgery, Bristol Heart Institute, Translational Health Sciences, University of Bristol, UK; Department of Cardiac Surgery, Bristol Heart Institute, Translational Health Sciences, University of Bristol, UK; National Institute for Health Research Bristol Biomedical Research Centre, University Hospitals Bristol and Weston NHS Foundation Trust and University of Bristol, Bristol, UK; Department of Cardiac Surgery, Bristol Heart Institute, Translational Health Sciences, University of Bristol, UK

**Keywords:** Machine learning, Mortality prediction, Cardiac surgery, Risk stratification, Benchmarking

## Abstract

**OBJECTIVES:**

A study of the performance of in-hospital/30-day mortality risk prediction models using an alternative machine learning algorithm (XGBoost) in adults undergoing cardiac surgery.

**METHODS:**

Retrospective analyses of prospectively routinely collected data on adult patients undergoing cardiac surgery in the UK from January 2012 to March 2019. Data were temporally split 70:30 into training and validation subsets. Independent mortality prediction models were created using sequential backward floating selection starting with 61 variables. Assessments of discrimination, calibration, and clinical utility of the resultant XGBoost model with 23 variables were then conducted.

**RESULTS:**

A total of 224,318 adults underwent cardiac surgery during the study period with a 2.76% (*N* = 6,100) mortality. In the testing cohort, there was good discrimination (area under the receiver operator curve 0.846, F1 0.277) and calibration (especially in high-risk patients). Decision curve analysis showed XGBoost-23 had a net benefit till a threshold probability of 60%. The most important variables were the type of operation, age, creatinine clearance, urgency of the procedure and the New York Heart Association score.

**CONCLUSIONS:**

Feature-selected XGBoost showed good discrimination, calibration and clinical benefit when predicting mortality post-cardiac surgery. Prospective external validation of a XGBoost-derived model performance is warranted.

## Graphical abstract

**Figure:**
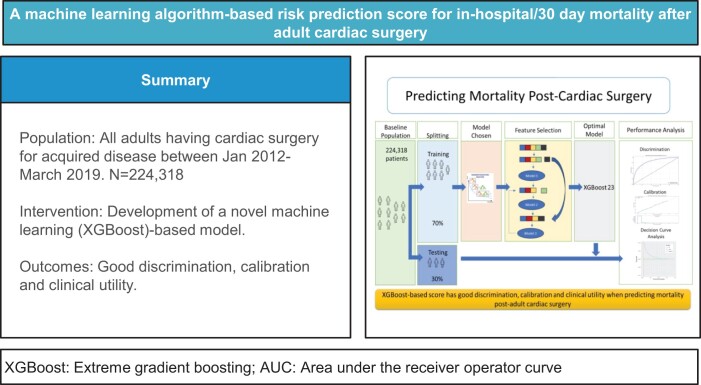

## INTRODUCTION

Prediction models are used in international guidelines [1, 2] to determine the most appropriate treatments and enable clinicians to counsel patients. With reductions in mortality, the emergence of minimally invasive surgery and interventional procedures, updated and more accurate models are needed. Accurate predictions also help in benchmarking individual surgical and institutional results. Older models, such as the logistic European System for Cardiac Operative Risk Evaluation (EuroSCORE) [3] and EuroSCOREII [4], need to be updated. The primary limitation of previous models is poor calibration with over-estimation of risk in the highest risk group [5–10]. Their performance varies by era, centre [4, 11–13] and inherent procedural risk [5, 14, 15], and the application of risk-adjusted mortality ratios is recommended [4, 12].

Current models utilize logistic regression (LR). Complex interactions may exist between the input variables and would need to be accounted for by the model developer. Alternative machine learning (ML) models use algorithms to interpret these interactions using large volumes of data. We have previously shown that alternative ML-based models had statistically better discrimination and clinical utility than a retrained-LR model but similar calibration when using the EuroSCOREII [11] variables. The most consistent improvement in performance was with a XGBoost-based model.

Herein, we have built upon our previous work by utilising all available variables routinely collected in the UK National Adult Cardiac Surgery Audit (NACSA). It is the largest dataset reviewed and utilizes an exhaustive variable selection approach in order to create a novel XGBoost-based model.

## METHODS

### Ethics statement

This register-based cohort study is part of research approved by the Health Research Authority and Health and Care Research Wales and a need for patients consent was waived (IRAS ID: 257758, 23.7.2019). Reporting of results follows the TRIPOD statement.

### Data extraction

A complete extract of prospectively collected data from the NACSA was obtained from the National Institute of Cardiovascular Outcomes Research (NICOR), containing data on all adults undergoing cardiac surgery in England and Wales between January 2012 and March 2019. Patients undergoing transplant, congenital surgery or isolated mechanical support insertion were excluded. The outcome measure was death within the same admission or 30 days of the index operation. Cases where the outcome measure was missing were excluded. Data processing and imputation of missing data were as previously described using R v4.0.2 [16]: ‘age and weight were imputed using the median and the absence of other variables was presumed to be an absence of that risk factor, a method previously established by NACSA’ [17, 18]. Assumptions and pre-processing of data were in line with previously published NACSA guidelines [11, 17, 18]. Missing data were <1%.

The variables routinely collected by NACSA were reviewed by 2 clinicians and a data analyst. Based on published literature, clinical experience and degree of missingness of the data 71 variables were initially reviewed. Those variables representing singular episodes were removed. The variables for pre-existing cardiac rhythm and the need for mechanical support were combined as shown in Supplementary Material, Fig. S1. Ultimately 61 variables were utilized (Supplementary Material, Fig. S1). These variables are defined in the NACSA database v5.1.0 [19].

### Statistical analysis

Categorical variables were summarized as counts and percentages. Continuous variables were summarized as mean and standard deviation.

### Model development

Both models were developed with Python [20] Scikit-learn v0.23.1 and Keras v2.4.0. Categorical variables’ values were converted to either binary values for dichotomous categories or the ordinal scale for multi-category variables. For reasons stated previously [11], we temporally split the data—70% of records (1 January 2012 to 31 December 2016) for training/validation and 30% (1 January 2017 to 31 March 2019) for testing. Data standardization was performed by subtracting variable mean and dividing by the standard deviation.

The XGBoost model is a combination of multiple short decision tree-based predictions. At each iteration, the performance of the model and the difference from the observed outcome is assessed. A weak decision tree model is then generated to predict this residual. This weaker tree is weighted by its predictive accuracy and added to the pre-existing model. Following multiple iterations, a final model is developed and used for onward assessment. Thus, it is an ensemble model that does not rely solely on one tree. It is a form of gradient boosting that incorporates a regularization term and cross-validation. The combination of these features in such a large dataset as ours reduces the risk of over-fitting. The final hyperparameters are listed in Supplementary Material, Table S2.The XGBoost-based model was further refined using a wrapper-based greedy algorithm for feature selection called sequential backward floating selection, using the mlxtend [21, 22] package in python. A model was developed using all 61 variables. Thereafter, sequentially one variable is removed at a time and the performance of the model assessed, in this case by measuring the area under the receiver operator curve (AUC). The ‘floating’ component allows the inclusion of variables even after the initial exclusion phase so long as the final combination of variables has an improved overall performance. This continues till the optimal combination of variables is reached.

Model validation and evaluation were conducted as before [11]. The primary outcomes were model discrimination (AUC, F1 score), calibration [visual inspection of a plot of observed versus expected outcomes and quantification of the observed minus expected outcomes (O-E), expected calibration error (ECE)], Brier score and clinical utility [decision curve analysis (DCA)]. 5-fold cross-validation was applied for evaluation of the training/validation set for AUC, F1, O-E and net benefit (NB) with the corresponding 95% confidence intervals (CIs) calculated using bootstrap-t sampling (*n* = 100) with replacement. The latter emphasizes the true positives and penalizes a high type II error rate. The calculation and interpretation of these are stated in our previous study [11]. An ideal model has a high AUC and F1 score, be on the bisector of the observed-to-expected curve, have a high adjusted ECE/Brier score (1−ECE and 1−Brier) and a high overall NB (i.e. the higher the curve on the DCA plot the better the clinical performance).

### Variable importance

The SHapley Adaptive exPlanations (SHAP) method [23] was used to understand variable importance in the final model. SHAP values [24] are derived from game theory and assign an importance value to each variable. In the final model, a prediction is made for an individual in the presence or absence of a given variable. The difference between the predictions is the marginal contribution of the variable to the prediction. Differing combinations of variables are utilized and averaged to determine the final SHAP value of a given variable and hence its importance to the final model.

## RESULTS

### Demographics

**Figure 1:**
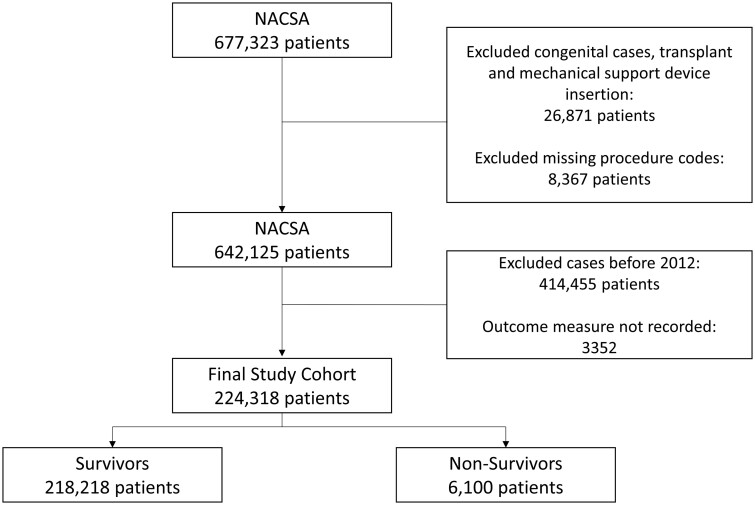
Consort diagram showing flow of participants through the study. NACSA: National Adult Cardiac Surgery Audit.

A total of 224 318 adults underwent cardiac surgery across 42 centres during the study period. There were 6100 deaths (2.72%) (Fig. 1). Baseline differences in the variables between survivors and non-survivors are shown in Table 1 and Supplementary Material, Table S1.

**Table 1:** Baseline patient demographics

Characteristic	Overall	Survivor	Nonsurvivor	Difference^b^	95% CI	*P*-value^b^
	*N* = 224 318^a^	*N* = 218 218^a^	*N* = 6100^a^			
Age (years)						<0.001
Median (IQR)	69.30 (61.10–76.00)	69.20 (61.00–75.90)	73.30 (65.60–78.80)			
Weight						<0.001
Median (IQR)	81.00 (71.00–92.00)	81.00 (71.00–92.00)	77.00 (66.00–90.00)			
BMI						<0.001
Median (IQR)	27.68 (25.10–31.19)	27.70 (25.14–31.20)	27.61 (24.22–30.84)			
Female	60 368/224 318 (27%)	58 102/218 218 (27%)	2266/6100 (37%)	−11%	−12% to −9.3%	<0.001
Hypertension	155 736/224 318 (69%)	151 386/218 218 (69%)	4350/6100 (71%)	−1.9%	−3.1% to −0.78%	0.001
Diabetes						0.199
Not diabetic	169 435/224 318 (76%)	164 918/218 218 (76%)	4517/6100 (74%)			
Diet controlled	8836/224 318 (3.9%)	8571/218 218 (3.9%)	265/6100 (4.3%)			
Oral therapy	32 844/224 318 (15%)	31 969/218 218 (15%)	875/6100 (14%)			
Insulin-dependent diabetes	13 203/224 318 (5.9%)	12 760/218 218 (5.8%)	443/6100 (7.3%)			
Smoker						<0.001
Never smoked	94 188/224 318 (42%)	91 567/218 218 (42%)	2621/6100 (43%)			
Ex-smoker	107 027/224 318 (48%)	104 146/218 218 (48%)	2881/6100 (47%)			
Current smoker	23 103/224 318 (10%)	22 505/218 218 (10%)	598/6100 (9.8%)			
History of pulmonary disease	27 585/224 318 (12%)	26 401/218 218 (12%)	1184/6100 (19%)	−7.3%	−8.3% to −6.3%	<0.001
History of stroke						<0.001
TIA	9616/218 634 (4.4%)	9237/212 748 (4.3%)	379/5886 (6.4%)			
CVA with full recovery	5122/218 634 (2.3%)	4901/212 748 (2.3%)	221/5886 (3.8%)			
CVA with residual neurological deficit	3384/218 634 (1.5%)	3181/212 748 (1.5%)	203/5886 (3.4%)			
(Missing)	5684	5470	214			
Neurologic dysfunction	7141/224 318 (3.2%)	6743/218 218 (3.1%)	398/6100 (6.5%)	−3.4%	−4.1% to −2.8%	<0.001
Peripheral vascular disease	23 462/224 318 (10%)	22 264/218 218 (10%)	1198/6100 (20%)	−9.4%	−10% to −8.4%	<0.001
Poor mobility	1935/224 318 (0.9%)	1798/218 218 (0.8%)	137/6100 (2.2%)	−1.4%	−1.8% to −1.0%	<0.001
Pulmonary hypertension	4651/224 318 (2.1%)	4249/218 218 (1.9%)	402/6100 (6.6%)			<0.001
LV function						<0.001
Very poor	1382/224 318 (0.6%)	1246/218 218 (0.6%)	136/6100 (2.2%)			
Poor	4512/224 318 (2.0%)	4207/218 218 (1.9%)	305/6100 (5.0%)			
Fair	31 444/224 318 (14%)	30 372/218 218 (14%)	1072/6100 (18%)			
Good	186 980/224 318 (83%)	182 393/218 218 (84%)	4587/6100 (75%)			
Creatinine						
Median (IQR)	85.00 (73.00–101.00)	85.00 (73.00–100.00)	100.00 (80.00–131.00)			
Mean (SD)	93.99 (50.43)	93.22 (48.76)	122.49 (88.44)	−29	−32 to −27	<0.001
(Missing)	14 266	13 691	575			
Creatinine clearance						<0.001
Median (IQR)	82.57 (62.67–100.58)	82.95 (63.18–100.84)	66.29 (46.85–99.04)			
(Missing)	33 678	32 990	688			
Pre-op dialysis						<0.001
None	214 727/220 015 (98%)	209 500/214 122 (98%)	5227/5893 (89%)			
Dialysis for acute renal failure within 6 weeks of surgery	838/220 015 (0.4%)	687/214 122 (0.3%)	151/5893 (2.6%)			
Dialysis for chronic renal failure	1656/220 015 (0.8%)	1484/214 122 (0.7%)	172/5893 (2.9%)			
No dialysis but acute renal failure preop	2794/220 015 (1.3%)	2451/214 122 (1.1%)	343/5893 (5.8%)			
(Missing)	4303	4096	207			
CCS						<0.001
0	75 514/224 318 (34%)	72 859/218 218 (33%)	2655/6100 (44%)			
1	21 249/224 318 (9.5%)	20 756/218 218 (9.5%)	493/6100 (8.1%)			
2	64 613/224 318 (29%)	63 575/218 218 (29%)	1038/6100 (17%)			
3	43 717/224 318 (19%)	42 715/218 218 (20%)	1002/6100 (16%)			
4	19 225/224 318 (8.6%)	18 313/218 218 (8.4%)	912/6100 (15%)			
NYHA						<0.001
1	48 602/224 318 (22%)	47 578/218 218 (22%)	1024/6100 (17%)			
2	97 609/224 318 (44%)	96 017/218 218 (44%)	1592/6100 (26%)			
3	65 752/224 318 (29%)	63 556/218 218 (29%)	2196/6100 (36%)			
4	12 355/224 318 (5.5%)	11 067/218 218 (5.1%)	1288/6100 (21%)			
Urgency						<0.001
Elective	142 456/224 318 (64%)	140 024/218 218 (64%)	2432/6100 (40%)			
Urgent	73 575/224 318 (33%)	71 455/218 218 (33%)	2120/6100 (35%)			
Emergency	7395/224 318 (3.3%)	6234/218 218 (2.9%)	1161/6100 (19%)			
Salvage	892/224 318 (0.4%)	505/218 218 (0.2%)	387/6100 (6.3%)			
Number of previous MIs						<0.001
0	152 138/224 318 (68%)	148 288/218 218 (68%)	3850/6100 (63%)			
1	61 800/224 318 (28%)	60 000/218 218 (27%)	1800/6100 (30%)			
2	10 380/224 318 (4.6%)	9930/218 218 (4.6%)	450/6100 (7.4%)			
Recent MI	44 651/224 318 (20%)	43 141/218 218 (20%)	1510/6100 (25%)	−5.0%	−6.1% to −3.9%	<0.001
Interval from MI to surgery						<0.001
No previous MI	153 246/224 318 (68%)	149 354/218 218 (68%)	3892/6100 (64%)			
MI < 6 hours	516/224 318 (0.2%)	409/218 218 (0.2%)	107/6100 (1.8%)			
MI 6–24 hours	1023/224 318 (0.5%)	880/218 218 (0.4%)	143/6100 (2.3%)			
MI 1–30 days	36 296/224 318 (16%)	35 222/218 218 (16%)	1074/6100 (18%)			
MI 31–90 days	6816/224 318 (3.0%)	6630/218 218 (3.0%)	186/6100 (3.0%)			
MI >90 days	26 421/224 318 (12%)	25 723/218 218 (12%)	698/6100 (11%)			
Previous PCI						<0.001
No previous PCI	198 924/224 318 (89%)	193 587/218 218 (89%)	5337/6100 (87%)			
PCI <24 h before surgery	931/224 318 (0.4%)	805/218 218 (0.4%)	126/6100 (2.1%)			
PCI >24 h before surgery same admission	2067/224 318 (0.9%)	1958/218 218 (0.9%)	109/6100 (1.8%)			
PCI >24 h before surgery previous admission	22 396/224 318 (10.0%)	21 868/218 218 (10%)	528/6100 (8.7%)			
Preoperative AF	24 923/224 318 (11%)	23 637/218 218 (11%)	1286/6100 (21%)	−10%	−11% to −9.2%	<0.001
Preoperative VF or VT	440/224 318 (0.2%)	387/218 218 (0.2%)	53/6100 (0.9%)	−0.69%	−0.93% to −0.45%	<0.001
Preoperative CHB/paced	3203/224 318 (1.4%)	2999/218 218 (1.4%)	204/6100 (3.3%)	−2.0%	−2.4% to −1.5%	<0.001
Left main stem disease	38 474/224 318 (17%)	37 529/218 218 (17%)	945/6100 (15%)	1.7%	0.78%,2.6%	<0.001
Systolic PA pressure				−11	−12 to −9.9	<0.001
Median (IQR)	24.00 (0.00–35.00)	23.00 (0.00–35.00)	32.00 (10.00–54.00)			
Mean (SD)	23.30 (21.44)	22.93 (21.20)	33.84 (25.28)			
(Missing)	153 722	150 070	3652			
Critical preoperative state	10 355/224 318 (4.6%)	8918/218 218 (4.1%)	1437/6100 (24%)	−19%	−21% to −18%	<0.001
Cardiogenic shock	2943/224 318 (1.3%)	2204/218 218 (1.0%)	739/6100 (12%)	−11%	−12% to −10%	<0.001
Preoperative ventilation	1838/224 318 (0.8%)	1399/218 218 (0.6%)	439/6100 (7.2%)	−6.6%	−7.2% to −5.9%	<0.001
Preoperative nitrates	10 208/224 318 (4.6%)	9551/218 218 (4.4%)	657/6100 (11%)	−6.4%	−7.2% to −5.6%	<0.001
Preoperative inotropes	2651/224,31 (1.2%)	1994/218218 (0.9%)	657/6100 (11%)	−9.9%	−11% to −9.1%	<0.001
Number of previous operations						<0.001
0	154 514/163 933 (94%)	150 780/159 244 (95%)	3734/4689 (80%)			
1	8372/163933 (5.1%)	7555/159 244 (4.7%)	817/4689 (17%)			
2	877/163 933 (0.5%)	773/159 244 (0.5%)	104/4689 (2.2%)			
3	140/163 933 (<0.1%)	115/159 244 (<0.1%)	25/4689 (0.5%)			
4	27/163 933 (<0.1%)	19/159 244 (<0.1%)	8/4689 (0.2%)			
5	1/163 933 (<0.1%)	1/159 244 (<0.1%)	0/4689 (0%)			
6	2/163 933 (<0.1%)	1/159 244 (<0.1%)	1/4689 (<0.1%)			
(Missing)	60 385	58 974	1411			
Previous CABG	3010/224 318 (1.3%)	2666/218 218 (1.2%)	344/6100 (5.6%)	−4.4%	−5.0% to −3.8%	<0.001
Previous valve surgery	6360/224 318 (2.8%)	5695/218 218 (2.6%)	665/6100 (11%)	−8.3%	−9.1% to −7.5%	<0.001
Previous aortic arch surgery	1223/224 318 (0.5%)	1050/218 218 (0.5%)	173/6100 (2.8%)	−2.4%	−2.8% to −1.9%	<0.001
Previous descending aortic surgery	1/224 318 (<0.1%)	1/218 218 (<0.1%)	0/6100 (0%)	0.00%	0.00% to 0.00%	>0.9
Previous thoracic surgery	1/224 318 (<0.1%)	1/218 218 (<0.1%)	0/6100 (0%)	0.00%	0.00% to 0.00%	>0.9
Endocarditis	6309/224 318 (2.8%)	5816/218 218 (2.7%)	493/6100 (8.1%)	−5.4%	−6.1% to −4.7%	<0.001
Post-infarct VSD	195/224 318 (<0.1%)	111/218 218 (<0.1%)	84/6100 (1.4%)	−1.3%	−1.6% to −1.0%	<0.001
Operation						<0.001
CABG only	113,190 / 224,318 (50.5%)	111 640/218 218 (51.2%)	1550/6100 (25.4%)	1,550 / 6,100 (25.4%)		
AVR only	35,161 / 224,318 (15.7%)	34 570/218 218 (15.8%)	591/6100 (9.7%)	591 / 6,100 (9.7%)		
CABG+AVR	21,978 / 224,318 (9.8%)	21 187/218 218 (9.7%)	791/6100 (13%)	791 / 6,100 (13%)		
CABG+MVR	4,556 / 224,318 (2.0%)	4268/218 218 (2%)	288/6100 (4.7%)	288 / 6,100 (4.7%)		
MVR only	14,005 / 224,318 (6.2%)	13 671/218 218 (6.3%)	334/6100 (5.5%)	334 / 6,100 (5.5%)		
Aortic ± other	13,922 / 224,318 (6.2%)	12 669/218 218 (5.8%)	1253/6100 (20.5%)	1,253 / 6,100 (20.5%)		
Other	18,598 / 224,318 (8.3%)	17 514/218 218 (8.0%)	1084/6100 (17.8%)	1,084 / 6,100 (17.8%)		
Unclassified	2,878 / 224,318 (1.3%)	2699/218 218 (1.2%)	209/6100 (3.4%)	209 / 6,100 (3.4%)		
Days between angiogram and surgery						0.2
Median (IQR)	62.00 (14.00–136.04)	62.00 (14.00–136.04)	36.00 (9.00–137.00)			
(Missing)	68 444	66 012	2432			
Left main stem disease	38 474/224 318 (17%)	37 529/218 218 (17%)	945/6100 (15%)	1.7%	0.78%,2.6%	<0.001
Extent of CAD						
Nil > 50%	61 174/200 018 (31%)	59 487/195 121 (30%)	1687/4897 (34%)			
One vessel >50%	18 092/200 018 (9.0%)	17 510/195 121 (9.0%)	582/4 897 (12%)			
2 vessels >50%	31 917/200 018 (16%)	31 206/195 121 (16%)	711/4897 (15%)			
3 vessels > 50%	88 835/200 018 (44%)	86 918/195 121 (45%)	1917/4897 (39%)			
(Missing)	24 300	23 097	1203			
Number of grafts						<0.001
1	19 083/164 803 (12%)	18 320/160 736 (11%)	763/4067 (19%)			
2	35 308/164 803 (21%)	34 428/160 736 (21%)	880/4067 (22%)			
3	61 855/164 803 (38%)	60 648/160 736 (38%)	1207/4067 (30%)			
4	25 362/164 803 (15%)	24 965/160 736 (16%)	397/4067 (9.8%)			
5	3362/164 803 (2.0%)	3294/160 736 (2.0%)	68/4067 (1.7%)			
6	339/164 803 (0.2%)	331/160 736 (0.2%)	8/4067 (0.2%)			
(Missing)	59 515	57 482	2033			
First operator grade						<0.001
Consultant	170 813/211 897 (81%)	165 411/205 977 (80%)	5402/5920 (91%)			
Staff grade	6745/211 897 (3.2%)	6674/205 977 (3.2%)	71/5920 (1.2%)			
Registrar	30 167/211 897 (14%)	29 840/205 977 (14%)	327/5920 (5.5%)			
SHO	4172/211 897 (2.0%)	4052/205 977 (2.0%)	120/5920 (2.0%)			
(Missing)	12 421	12 241	180			
Payer status						<0.001
NHS	214 259/222 167 (96%)	208 318/216 124 (96%)	5941/6043 (98%)			
Private	7908/222 167 (3.6%)	7806/216 124 (3.6%)	102/6043 (1.7%)			
(Missing)	2151	2094	57			

a Mean (SD) or frequency (%).

b Two sample test for equality of proportions; Welch 2-sample *t*-test.

BMI: basal metabolic index; CABG: coronary artery bypass graft; CCS: Canadian Cardiovascular score; CI: confidence interval; CVA: cerebrovascular accident, IQR: interquartile range; LV: left ventricular, MI: myocardial infarction; NYHA: New York Heart Association, TIA: transient ischaemic attack, AF: Atrial fibrillation, AVR: aortic valve repair/replacement, CAD: coronary artery disease, CHB: complete heart block, MVR: mitral valve repair/replacement, NHS: National Health Service, PA: pulmonary artery, PCI: percutaneous coronary intervention, SHO: senior house officer, VF: ventricular fibrillation, VSD: ventricular septal defect, VT: ventricular tachycardia.

### Final model

The final model consisted of the variables in Table 2.

**Table 2:** Variables used in the final model

XGBoost-23
Type of operation
Age
Creatinine clearance
Urgency
New York Heart Association Score
Critical pre-operative state
Peripheral vascular disease
Previous myocardial infarction
Pre-operative cardiac rhythm
Pulmonary disease
First operator grade
Diabetes
Basal metabolic index
Mitral valve procedure
Left ventricular function
Pre-operative stroke
Previous valve surgery
Aortic arch procedure
Previous coronary artery bypass
Need for mechanical support
Hospital Code
Previous operation
Number of grafts

### XGBoost-23

**Figure 2:**
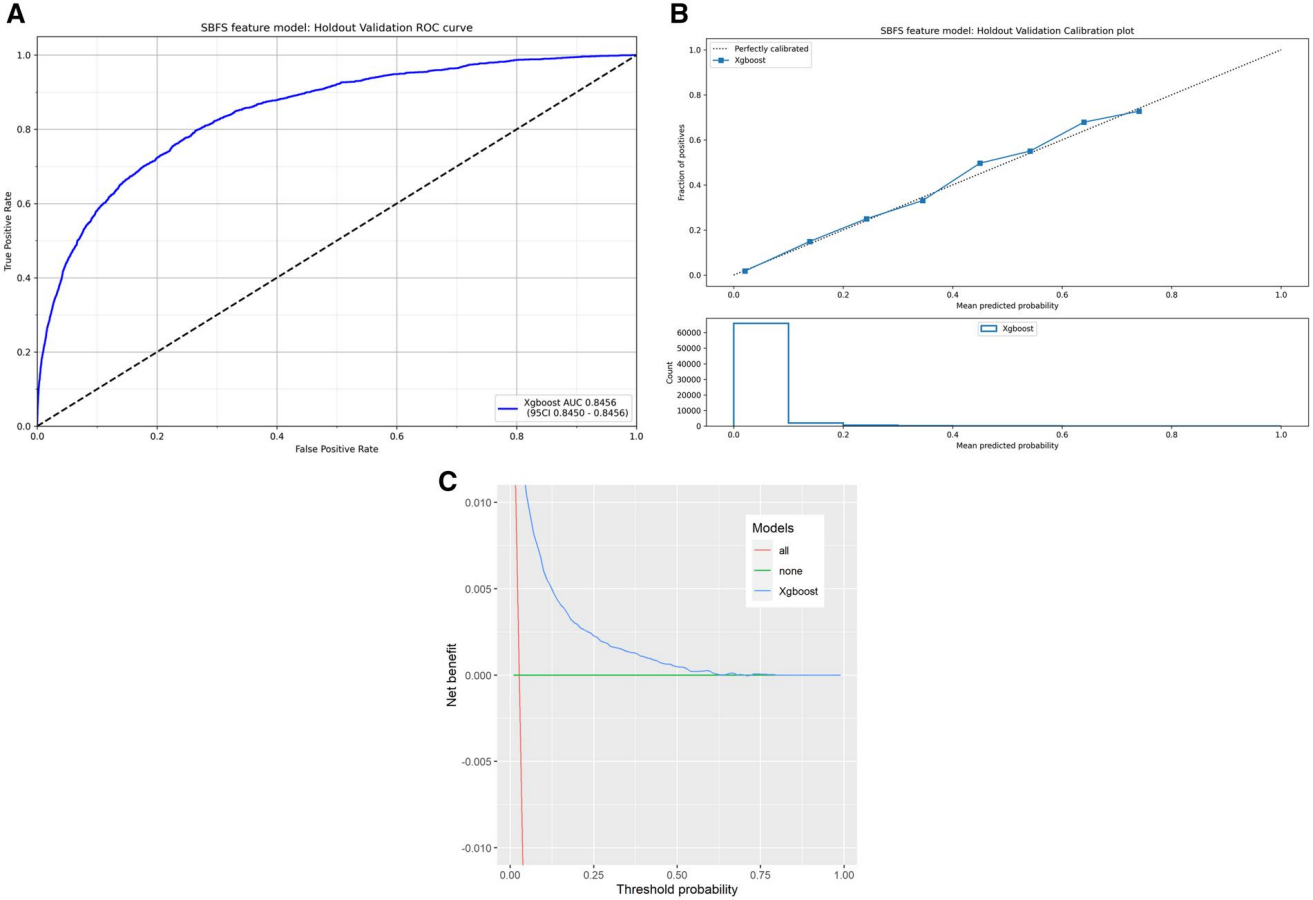
Performance of the final model developed using XGBoost with feature selection. (**A**) Discrimination, (**B**) calibration, (C) decision curve analysis. AUC: Area under the receiver operator curve; CI: confidence interval; ROC: receiver operating characteristic.

The XGBoost model with the greatest training set AUC had 27 variables—AUC 0.837 (95% CI: 0.837–0.838) and F1 score 0.277 (95% CI: 0.276–0.279) (Supplementary Material, Table S3). However, visual inspection of the AUC curve (Supplementary Material, Fig. S4) and output values (Supplementary Material, Table S3) showed that there was minimal loss in discrimination with less variables. Hence, after discussion and in the interest of parsimony, a ‘sensitivity’ analysis was performed to assess the alteration in model performance with a lower number of variables (20, 23, and 25) (Supplementary Material, Table S3). There was a marked drop in NB (treated) when going from 23 to 20 variables. There was risk over-estimation at 20 variables and under-estimation at 23 and 25 variables, with minimal differences in clinical utility between the 23 and 25 variable models. Good performance was achieved in terms of AUC, F1 score, Adjusted Brier Score, Adjusted ECE score and clinical NB when benchmarked against EuroSCORE II [25, 26] (Supplementary Material, Table S3B). On balance we opted to move forward with a XGBoost model with 23 variables (i.e. XGBoost-23)—AUC 0.846 (95% CI: 0.845–0.846) and F1 score 0.288 (95% CI: 0.287–0.290) (Fig. 2A), which showed good calibration even with those at high predicted risk (Fig. 2B). The number of patients in each risk centile, as predicted by XGBoost-23, are shown in Table 3 and Supplementary Material, Fig. S5. There are very few patients with a predicted risk above 30% that have undergone surgery, as would be expected. The DCA (Fig. 2C) showed an NB in those treated at all threshold probabilities below 60%.

**Table 3:** Number of patients in each decile of predicted risk, as per XGBoost-23, in the test group

Predicted risk range %	Total number	Survived	Not survived	Actual % risk
0–10%	65 868	64 731	1137	1.73
>10–20%	2061	1749	312	15.14
>20–30%	621	473	148	23.83
>30–40%	235	146	89	37.87
>40–50%	133	69	64	48.12
>50–60%	84	38	46	54.76
>60–70%	43	15	28	65.12
>70–80%	9	2	7	77.78
>80–90%	0	0	0	0.00
>90–100%	0	0	0	0.00

### Variable importance

**Figure 3:**
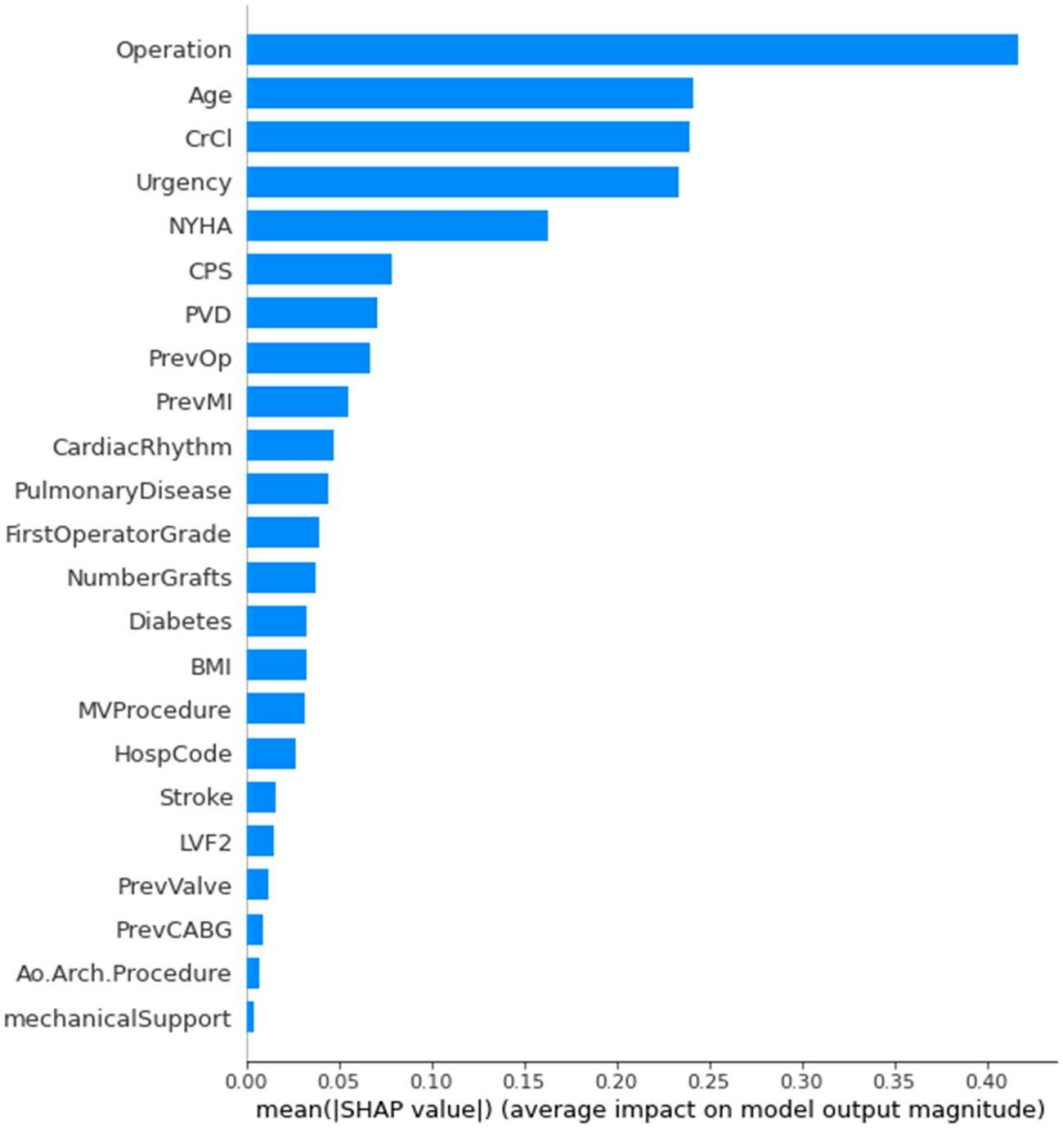
Variable importance of the final XGBoost-23 model. Ao.Arch.Procedure: operation on the aortic arch; BMI: basal metabolic index; CardiacRhythm: preoperative cardiac rhythm; CPS: critical pre-operative state; CrCl: creatinine clearance; HospCode: hospital identification code; LVF2: left ventricular function using same criteria as EuroSCOREII; mechanicalSupport: need for pre-operative mechanical support. NYHA: New York Heart Association Score; PrevCABG: previous coronary artery bypass grafts; PrevMI: number of previous myocardial infarctions; PrevOp: any previous cardiac surgery; PrevValve: previous valve surgery; PVD: peripheral vascular disease; Stroke: pre-operative stroke.

Variable importance of the XGBoost-23 model conducted on the training/validation set is shown in Fig. 3 and Supplementary Material, Table S4.The most influential factors are the type of operation, age, creatinine clearance, urgency and New York Heart Association scores.

## DISCUSSION

This study is an example of how using a feature-selected gradient boosting ML algorithm (XGBoost) can produce a mortality risk prediction model with good discrimination, calibration and clinical utility. This is the largest national study and builds on our previous work [11].

We began with 61 variables and used automated feature selection to develop the final model. It was developed with a view to optimize discrimination and indeed had not only a higher AUC and F1 score compared to EuroSCORE II and previous studies but also showed improved calibration and clinical utility [26]. It showed good calibration across all deciles of risk. Most patients had risks less than 10%, as would be expected of those undergoing surgery. However, in the higher-risk patients, XGBoost-23 continued to perform well. DCA showed that with probability thresholds under 60% there is a clear NB when using XGBoost-23 suggesting patients would be appropriately operated upon if using this model. Improved performance in the few high-risk patients becomes important when considering operations on patients with few alternatives (e.g. aortic dissections) or in units routinely operating on high-risk cases. It would be interesting to review model performance in those turned down for surgery but this data is not routinely collected.

Notably, we have not sought any information that is not already routinely gathered though many additional risk factors have been proposed previously [27, 28]. We also rationalized the final XGBoost model in the name of parsimony and to encourage its use by clinicians going forward.

No matter which modelling tool is utilized the relationship between patient, perioperative factors and death is inherently complex. Alternative ML models, such as XGBoost, offer the benefit of requiring little input from the model developer and having innate additive properties such that additional information can be used to adapt models without re-running the algorithm from the beginning. However, it is hard to depict as a standard formula and is less trusted by some. With the best performing model (XGBoost-23) we have described variable importance, which should also aid in greater understanding and acceptance in the wider surgical community. We are developing a website to host the model and allow prospective evaluation by others. LR has the advantage that it is well understood by the scientific community and publishing of coefficients allows external validation. However, modelling of interactions and non-linear relationships between the explanatory and response variables will be needed to optimize a LR model. The addition of new results would also require re-running the model development process.

Ensemble tree based, particularly gradient boosting, algorithms have been used with increasing frequency recently. Ours is the largest database studied, with only 3 other papers having more than 10 000 patients [30–32]. Most studies have shown marginal improvements in model performance with the use of alternative ML algorithms compared to commonly used models and retrained-LR models [31]. Interestingly Ong et al. [13] also showed improvements in discrimination when training models on local data. Although intuitive it reinforces the need to use either local variables or, as we have done, use hospital codes. Previous reviews have shown variable model performance on the basis of the hospital [4, 11–13] and operation undertaken [5, 14, 15]. Though the team behind the EuroSCORE advocated using hospital risk-adjusted mortality ratios, we felt that the hospital code could be incorporated into the final model from the outset and would increase ease of use. Zeng et al. [29] also performed a very thorough comparative study of different gradient boosting algorithms with a variety of feature selection methods but found only minor differences in performance between them and a retrained-LR model. Our study is unique in performing DCA in addition to discrimination and calibration. We feel this is crucial as improved clinical utility is the ultimate aim for prediction modelling.

Some variables are similar to those already taken into account in published scoring systems–age, creatinine clearance, New York Heart Association, urgency, critical pre-operative state, previous surgery, left ventricular function, peripheral vascular disease and pulmonary disease. Others have been replaced with similar variables of greater granularity, such as all diabetes management strategies, types of operations performed and distinguishing between mitral valve repair and replacement. New risk factors include the presence of a previous myocardial infarction (MI) not just recent MI, number of grafts inserted, pre-operative cardiac rhythm, first operator grade, basal metabolic index, pre-operative stroke, aortic arch procedure and need for mechanical support. Furthermore, differences in risk between those with previous valve surgery and previous coronary artery bypass surgery are also accounted for.

With the advent of more artificial intelligence-based search engines, there is increased public awareness and acceptance of these processes. We believe this should extend to the cardiac surgical community. With increasing server capacity and ultimately prospective data analysis we hope to be able to assess and fine tune this model further. The importance of risk prediction for morbidities (e.g. stroke, MI), resource utilization (e.g. length of stay, cost of intervention [33]) and interventional procedures [34] also has clear benefits for patient counselling and resource planning. Any future model utilized needs to be accurate, easy to use, avoid over-fitting and be recalibrated periodically.

### Limitations and future work

The current model was created using a historic database. With the advent of more interventional strategies and the interruption of services secondary to the pandemic we should explore temporal differences in model performance. Prospective analysis is warranted before routine adoption of any model. Mortality following cardiac surgery in the UK is low [35] and strategies for managing class imbalance are needed. Future work will also include the development of an optimized LR-based model in order to perform a comparative analysis.

## CONCLUSIONS

A feature-selected XGBoost-based model for 30 day or in-hospital mortality in adult cardiac surgery showed good discrimination, calibration and clinical benefit. Prospective assessment of a XGBoost-derived model in an external dataset is warranted.

## Supplementary Material

ezae368_Supplementary_Data

## Data Availability

The data underlying this article were provided by NICOR/HQIP under licence/by permission. Data will be shared on request to the corresponding author with permission of NICOR/HQIP.

## ABBREVATIONS

AUC: Area under the receiver operator curve
CI: Confidence interval
DCA: Decision curve analysis
ECE: Expected calibration error
EuroSCORE: European System for Cardiac Operative Risk Evaluation
LR: Logistic regression
MI: Myocardial infarction
ML: Machine learning
NACSA: National Adult Cardiac Surgery Audit
NB: Net benefit
SHAP: SHapley Adaptive exPlanations
XGBoost: Extreme gradient boosting machine
