# Supplementary material for: A machine learning algorithm-based risk prediction score for in-hospital/30-day mortality after adult cardiac surgery
Source: Eur J Cardiothorac Surg. 2024 Oct 7;66(4):ezae368. doi: 10.1093/ejcts/ezae368 (PMC11522872; doi:10.1093/ejcts/ezae368)

**Supplemental Material**

**A machine learning algorithm risk prediction score** **for in-hospital/30 day mortality in adult cardiac surgery**

Shubhra Sinha MBBS^1*^,Tim Dong^1*^MSc, Arnaldo Dimagli^1^ MD, Andy Judge PhD^1,2^ and Gianni D Angelini MD^1^.

References:

31. Betts KS, Marathe SP, Chai K, Konstantinov I, Iyengar A, Suna J, et al. A machine learning approach to predicting 30-day mortality following paediatric cardiac surgery: findings from the Australia New Zealand Congenital Outcomes Registry for Surgery (ANZCORS). Eur J Cardio-thoracic Surg. 2023;64(2):3–8.

32. Penny-Dimri JC, Bergmeir C, Reid CM, Williams-Spence J, Perry LA, Smith JA. Tree-based survival analysis improves mortality prediction in cardiac surgery. Front Cardiovasc Med. 2023;10(July):1–8.

33. Zea-Vera R, Ryan CT, Navarro SM, Havelka J, Wall MJ, Coselli JS, et al. Development of a Machine Learning Model to Predict Outcomes and Cost After Cardiac Surgery. Ann Thorac Surg [Internet]. 2023;115(6):1533–42. Available from: https://doi.org/10.1016/j.athoracsur.2022.06.055

34. Feng X, Zhang C, Huang X, Liu J, Jiang L, Xu L, et al. Machine learning improves mortality prediction in three-vessel disease. Atherosclerosis. 2023;367(July 2022):1–7.

35. National Cardiac Audit Programme. NATIONAL ADULT CARDIAC SURGERY AUDIT (NACSA) 2022 Summary Report. 2022; Available from: https://www.nicor.org.uk/wp-content/uploads/2023/06/10633_NICOR-Annual-Summary_NACSA_v4.pdf

Supplementary Table 1. Procedure specific details

|  | | Demographics | |  | | |  |
| --- | --- | --- | --- | --- | --- | --- | --- |
| Characteristic | Overall, N = 224,318^1^ | Survivor, N = 218,218^1^ | Non-Survivor, N = 6,100^1^ | Difference^2^ | 95% CI^23^ | p-value^2^ |  |
| **Acute Aortic Syndrome** | 3,172 / 224,318 (1.4%) | 2,659 / 218,218 (1.2%) | 513 / 6,100 (8.4%) | -7.2% | -7.9%, -6.5% | <0.001 |  |
| **Impeller** |  |  |  |  |  | <0.001 |  |
| Pre-op | 36 / 185,501 (<0.1%) | 31 / 180,260 (<0.1%) | 5 / 5,241 (<0.1%) |  |  |  |  |
| Intra-op | 132 / 185,501 (<0.1%) | 89 / 180,260 (<0.1%) | 43 / 5,241 (0.8%) |  |  |  |  |
| Post-op | 20 / 185,501 (<0.1%) | 9 / 180,260 (<0.1%) | 11 / 5,241 (0.2%) |  |  |  |  |
| (Missing) | 38,817 | 37,958 | 859 |  |  |  |  |
| **VAD** |  |  |  |  |  | <0.001 |  |
| Pre-op | 62 / 184,339 (<0.1%) | 54 / 179,157 (<0.1%) | 8 / 5,182 (0.2%) |  |  |  |  |
| Intra-op | 49 / 184,339 (<0.1%) | 20 / 179,157 (<0.1%) | 29 / 5,182 (0.6%) |  |  |  |  |
| Post-op | 83 / 184,339 (<0.1%) | 37 / 179,157 (<0.1%) | 46 / 5,182 (0.9%) |  |  |  |  |
| (Missing) | 39,979 | 39,061 | 918 |  |  |  |  |
| **IABP** |  |  |  |  |  | <0.001 |  |
| Pre-op | 3,398 / 190,640 (1.8%) | 3,032 / 185,228 (1.6%) | 366 / 5,412 (6.8%) |  |  |  |  |
| Intra-op | 2,329 / 190,640 (1.2%) | 1,805 / 185,228 (1.0%) | 524 / 5,412 (9.7%) |  |  |  |  |
| Post-op | 3,039 / 190,640 (1.6%) | 2,272 / 185,228 (1.2%) | 767 / 5,412 (14%) |  |  |  |  |
| (Missing) | 33,678 | 32,990 | 688 |  |  |  |  |
| **Other Mechanical Support** | |  |  |  |  |  | <0.001 |
| Pre-op | | 105 / 184,319 (<0.1%) | 86 / 179,140 (<0.1%) | 19 / 5,179 (0.4%) |  |  |  |
| Intra-op | | 80 / 184,319 (<0.1%) | 38 / 179,140 (<0.1%) | 42 / 5,179 (0.8%) |  |  |  |
| Post-op | | 139 / 184,319 (<0.1%) | 49 / 179,140 (<0.1%) | 90 / 5,179 (1.7%) |  |  |  |
| (Missing) | | 39,999 | 39,078 | 921 |  |  |  |
| **Pump Case** | 201,479 / 216,104 (93%) | 195,789 / 210,162 (93%) | 5,690 / 5,942 (96%) | -2.6% | -3.1%, -2.1% | <0.001 |  |
| (Missing) | 8,214 | 8,056 | 158 |  |  |  |  |
| **Number of valves** |  |  |  |  |  | <0.001 |  |
| 0 | 124,568 / 224,318 (56%) | 122,036 / 218,218 (56%) | 2,532 / 6,100 (42%) |  |  |  |  |
| 1 | 88,253 / 224,318 (39%) | 85,495 / 218,218 (39%) | 2,758 / 6,100 (45%) |  |  |  |  |
| 2 | 10,436 / 224,318 (4.7%) | 9,738 / 218,218 (4.5%) | 698 / 6,100 (11%) |  |  |  |  |
| 3 | 1,051 / 224,318 (0.5%) | 941 / 218,218 (0.4%) | 110 / 6,100 (1.8%) |  |  |  |  |
| 4 | 10 / 224,318 (<0.1%) | 8 / 218,218 (<0.1%) | 2 / 6,100 (<0.1%) |  |  |  |  |
| **Aortic valve procedure** |  |  |  |  |  | <0.001 |  |
| Replacement | 68,133 / 224,318 (30%) | 65,779 / 218,218 (30%) | 2,354 / 6,100 (39%) |  |  |  |  |
| Repair | 3 / 224,318 (<0.1%) | 3 / 218,218 (<0.1%) | 0 / 6,100 (0%) |  |  |  |  |
| Repair with ring | 34 / 224,318 (<0.1%) | 33 / 218,218 (<0.1%) | 1 / 6,100 (<0.1%) |  |  |  |  |
| Repair without ring | 921 / 224,318 (0.4%) | 859 / 218,218 (0.4%) | 62 / 6,100 (1.0%) |  |  |  |  |
| Isolated commisurotoplasty | 14 / 224,318 (<0.1%) | 11 / 218,218 (<0.1%) | 3 / 6,100 (<0.1%) |  |  |  |  |
| Excision only | 6 / 224,318 (<0.1%) | 5 / 218,218 (<0.1%) | 1 / 6,100 (<0.1%) |  |  |  |  |
| Inspection only | 131 / 224,318 (<0.1%) | 129 / 218,218 (<0.1%) | 2 / 6,100 (<0.1%) |  |  |  |  |
| Unspecified | 2,952 / 224,318 (1.3%) | 2,863 / 218,218 (1.3%) | 89 / 6,100 (1.5%) |  |  |  |  |
| **Mitral valve procedure** |  |  |  |  |  | <0.001 |  |
| Nil | 192,152 / 224,318 (86%) | 187,512 / 218,218 (86%) | 4,640 / 6,100 (76%) |  |  |  |  |
| Replacement | 12,313 / 224,318 (5.5%) | 11,392 / 218,218 (5.2%) | 921 / 6,100 (15%) |  |  |  |  |
| Repair | 71 / 224,318 (<0.1%) | 68 / 218,218 (<0.1%) | 3 / 6,100 (<0.1%) |  |  |  |  |
| Repair with ring | 17,159 / 224,318 (7.6%) | 16,736 / 218,218 (7.7%) | 423 / 6,100 (6.9%) |  |  |  |  |
| Repair without ring | 841 / 224,318 (0.4%) | 802 / 218,218 (0.4%) | 39 / 6,100 (0.6%) |  |  |  |  |
| Isolated commissuroplasty | 11 / 224,318 (<0.1%) | 9 / 218,218 (<0.1%) | 2 / 6,100 (<0.1%) |  |  |  |  |
| Excision only | 18 / 224,318 (<0.1%) | 18 / 218,218 (<0.1%) | 0 / 6,100 (0%) |  |  |  |  |
| Inspection only | 72 / 224,318 (<0.1%) | 62 / 218,218 (<0.1%) | 10 / 6,100 (0.2%) |  |  |  |  |
| Unspecified | 1,681 / 224,318 (0.7%) | 1,619 / 218,218 (0.7%) | 62 / 6,100 (1.0%) |  |  |  |  |
| **Tricuspid valve procedure** |  |  |  |  |  | <0.001 |  |
| Nil | 216,564 / 224,318 (97%) | 210,958 / 218,218 (97%) | 5,606 / 6,100 (92%) |  |  |  |  |
| Replacement | 660 / 224,318 (0.3%) | 590 / 218,218 (0.3%) | 70 / 6,100 (1.1%) |  |  |  |  |
| Repair | 8 / 224,318 (<0.1%) | 8 / 218,218 (<0.1%) | 0 / 6,100 (0%) |  |  |  |  |
| Repair with ring | 5,754 / 224,318 (2.6%) | 5,408 / 218,218 (2.5%) | 346 / 6,100 (5.7%) |  |  |  |  |
| Repair without ring | 964 / 224,318 (0.4%) | 905 / 218,218 (0.4%) | 59 / 6,100 (1.0%) |  |  |  |  |
| Isolated commisuroplasty | 3 / 224,318 (<0.1%) | 3 / 218,218 (<0.1%) | 0 / 6,100 (0%) |  |  |  |  |
| Excision only | 6 / 224,318 (<0.1%) | 5 / 218,218 (<0.1%) | 1 / 6,100 (<0.1%) |  |  |  |  |
| Inspection only | 21 / 224,318 (<0.1%) | 20 / 218,218 (<0.1%) | 1 / 6,100 (<0.1%) |  |  |  |  |
| Unspecified | 338 / 224,318 (0.2%) | 321 / 218,218 (0.1%) | 17 / 6,100 (0.3%) |  |  |  |  |
| **Pulmonary valve procedure** |  |  |  |  |  | <0.001 |  |
| Nil | 224,114 / 224,318 (100%) | 218,040 / 218,218 (100%) | 6,074 / 6,100 (100%) |  |  |  |  |
| Replacement | 188 / 224,318 (<0.1%) | 163 / 218,218 (<0.1%) | 25 / 6,100 (0.4%) |  |  |  |  |
| Repair with ring | 1 / 224,318 (<0.1%) | 1 / 218,218 (<0.1%) | 0 / 6,100 (0%) |  |  |  |  |
| Repair without ring | 4 / 224,318 (<0.1%) | 4 / 218,218 (<0.1%) | 0 / 6,100 (0%) |  |  |  |  |
| Excision only | 1 / 224,318 (<0.1%) | 1 / 218,218 (<0.1%) | 0 / 6,100 (0%) |  |  |  |  |
| Inspection only | 5 / 224,318 (<0.1%) | 4 / 218,218 (<0.1%) | 1 / 6,100 (<0.1%) |  |  |  |  |
| Unspecified | 5 / 224,318 (<0.1%) | 5 / 218,218 (<0.1%) | 0 / 6,100 (0%) |  |  |  |  |
| **Aortic Root Procedure** |  |  |  |  |  | <0.001 |  |
| Interposition tube graft |  | 0 / 218,218 (0%) | 1 / 6,100 (0%) |  |  |  |  |
| Tube graft + separate AVR |  | 2 /218,218 (0%) | 0 / 6,100 (0%) |  |  |  |  |
| Root replacement with composite valve graft and coronary reimplantation (Modified Bentall or Cabroll) |  | 2,909/218,218 (1.3%) | 285 / 6,100 (4.7%) |  |  |  |  |
| Root replacement with preservation of native valve and coronary reimplantation |  | 628 / 218,218 (0.3%) | 23 / 6,100 (0.4%) |  |  |  |  |
| Homograft Root Replacement |  | 144 /218, 218 (0.1%) | 41 / 6,100 (0.7%) |  |  |  |  |
| Ross Procedure |  | 26 / 218,218 (0%) | 0 / 6,100 (0%) |  |  |  |  |
| Aortic patch graft |  | 7 / 218,218 (0%) | 0 / 6,100 (0%) |  |  |  |  |
| Sinus of Valsalva Repair |  | 404 / 218,218 (0.2%) | 37 / 6,100 (0.6%) |  |  |  |  |
| Reduction aortoplasty |  | 1 / 218,218 (0%) | 1 / 6,100 (0%) |  |  |  |  |
| **Ascending Aortic Procedure** |  |  |  |  |  | <0.001 |  |
| Interposition tube graft |  | 4,960 / 218,218 (2.3%) | 539 / 6,100 (8.8%) |  |  |  |  |
| Interposition tube graft with reimplantation of major vessels |  | 21 / 218,218 (0%) | 4 / 6,100 (0.1%) |  |  |  |  |
| Tube graft + separate AVR |  | 1,140 / 218,218 (0.5%) | 57 / 6,100 (0.9%) |  |  |  |  |
| Root replacement with composite valve graft and coronary reimplantation (Modified Bentall or Cabroll) |  | 1,450 / 218,218 (0.7%) | 124 / 6,100 (2%) |  |  |  |  |
| Root replacement with preservation of native valve and coronary reimplantation |  | 221 / 218,218 (0.1%) | 9 / 6,100 (0.1%) |  |  |  |  |
| Homograft Root Replacement |  | 37 /218,218 (0%) | 13 / 6,100 (0.2%) |  |  |  |  |
| Ross Procedure |  | 5 / 218,218 (0%) | 0 / 6,100 (0%) |  |  |  |  |
| Aortic patch graft |  | 220 / 218,218 (0.1%) | 24 / 6,100 (0.4%) |  |  |  |  |
| Sinus of Valsalva Repair |  | 0 / 218,218 (0%) | 1 / 6,100 (<0.1%) |  |  |  |  |
| **Aortic arch procedure** |  |  |  |  |  | <0.001 |  |
| Interposition tube graft | 33 / 224,318 (<0.1%) | 32 / 218,218 (<0.1%) | 1 / 6,100 (<0.1%) |  |  |  |  |
| Interposition tube graft with reimplantation of major vessels | 1,223 / 224,318 (0.5%) | 1,049 / 218,218 (0.5%) | 174 / 6,100 (2.9%) |  |  |  |  |
| Tube graft + separate AVR | 1 / 224,318 (<0.1%) | 1 / 218,218 (<0.1%) | 0 / 6,100 (0%) |  |  |  |  |
| Root replacement with composite valve graft and coronary reimplantation (Modified Bentall or Cabroll) | 2 / 224,318 (<0.1%) | 1 / 218,218 (<0.1%) | 1 / 6,100 (<0.1%) |  |  |  |  |
| Root replacement with preservation of native valve and coronary reimplantation | 1 / 224,318 (<0.1%) | 1 / 218,218 (<0.1%) | 0 / 6,100 (0%) |  |  |  |  |
| Aortic patch graft | 139 / 224,318 (<0.1%) | 115 / 218,218 (<0.1%) | 24 / 6,100 (0.4%) |  |  |  |  |
| Concomitant endovascular aortic procedure | 58 / 224,318 (<0.1%) | 49 / 218,218 (<0.1%) | 9 / 6,100 (0.1%) |  |  |  |  |
| Extra-anatomic bypass | 58 / 224,318 (<0.1%) | 50 / 218,218 (<0.1%) | 8 / 6,100 (0.1%) |  |  |  |  |
| **Descending aortic procedure** |  |  |  |  |  | <0.001 |  |
| Interposition tube graft | 311 / 224,318 (0.1%) | 274 / 218,218 (0.1%) | 37 / 6,100 (0.6%) |  |  |  |  |
| Aortic patch graft | 5 / 224,318 (<0.1%) | 3 / 218,218 (<0.1%) | 2 / 6,100 (<0.1%) |  |  |  |  |
| Concomitant endovascular aortic procedure | 8 / 224,318 (<0.1%) | 5 / 218,218 (<0.1%) | 3 / 6,100 (<0.1%) |  |  |  |  |
| Extra-anatomic bypass | 166 / 224,318 (<0.1%) | 140 / 218,218 (<0.1%) | 26 / 6,100 (0.4%) |  |  |  |  |
| **Abdominal aortic surgery** |  |  |  |  |  | <0.001 |  |
| Interposition tube graft | 178 / 224,318 (<0.1%) | 148 / 218,218 (<0.1%) | 30 / 6,100 (0.5%) |  |  |  |  |
| Interposition tube graft with reimplantation of major vessels | 76 / 224,318 (<0.1%) | 63 / 218,218 (<0.1%) | 13 / 6,100 (0.2%) |  |  |  |  |
| Concomitant endovascular aortic procedure | 1 / 224,318 (<0.1%) | 0 / 218,218 (0%) | 1 / 6,100 (<0.1%) |  |  |  |  |
| Extra-anatomic bypass | 6 / 224,318 (<0.1%) | 5 / 218,218 (<0.1%) | 1 / 6,100 (<0.1%) |  |  |  |  |
| **Median Sternotomy** | 222,832 / 224,318 (99%) | 216,764 / 218,218 (99%) | 6,068 / 6,100 (99%) | -0.14% | -0.33%, 0.05% | 0.2 |  |
| **Partial Sternotomy** | 586 / 224,318 (0.3%) | 581 / 218,218 (0.3%) | 5 / 6,100 (<0.1%) | 0.18% | 0.10%, 0.27% | 0.008 |  |
| **Thoracotomy** | 203 / 224,318 (<0.1%) | 190 / 218,218 (<0.1%) | 13 / 6,100 (0.2%) | -0.13% | -0.25%, 0.00% | 0.003 |  |
| **Mini-thoracotomy** | 615 / 224,318 (0.3%) | 606 / 218,218 (0.3%) | 9 / 6,100 (0.1%) | 0.13% | 0.02%, 0.24% | 0.073 |  |
| **Year** |  |  |  |  |  | 0.01 |  |
| 2012 | 31,317 / 224,318 (14%) | 30,362 / 218,218 (14%) | 955 / 6,100 (16%) |  |  |  |  |
| 2013 | 30,867 / 224,318 (14%) | 29,985 / 218,218 (14%) | 882 / 6,100 (14%) |  |  |  |  |
| 2014 | 31,288 / 224,318 (14%) | 30,432 / 218,218 (14%) | 856 / 6,100 (14%) |  |  |  |  |
| 2015 | 30,671 / 224,318 (14%) | 29,892 / 218,218 (14%) | 779 / 6,100 (13%) |  |  |  |  |
| 2016 | 31,121 / 224,318 (14%) | 30,324 / 218,218 (14%) | 797 / 6,100 (13%) |  |  |  |  |
| 2017 | 32,000 / 224,318 (14%) | 31,133 / 218,218 (14%) | 867 / 6,100 (14%) |  |  |  |  |
| 2018 | 29,934 / 224,318 (13%) | 29,157 / 218,218 (13%) | 777 / 6,100 (13%) |  |  |  |  |
| 2019 | 7,120 / 224,318 (3.2%) | 6,933 / 218,218 (3.2%) | 187 / 6,100 (3.1%) |  |  |  |  |
| ^1^Mean (SD) or Frequency (%) | | | | | | |  |
| ^2^Two sample test for equality of proportions; Welch Two Sample t-test | | | | | | |  |
| ^3^CI = Confidence Interval | | | | | | |  |

Supplementary Table 2. Hyperparameters for final XGBoost model

| Hyperparameter |  |
| --- | --- |
| Max_depth | 5 |
| Eta | 1 |
| objective | Binary logistic |
| subsample | 0.6 |
| Min_child_weight | 1 |
| gamma | 0.5 |
| Colsample_bytree | 0.8 |
| Learning_rate | 0.02 |
| N_estimators | 300 |

Supplementary Table 3.Quantitative Model Performance.Models developed using the sequential backward floating selection technique.95% confidence intervals in brackets.AUC: Area under the receiver operator curve; adjusted ECE: 1-Expected calibration error; adjusted brier score: 1- brier score.

1. Training/Validation Dataset

| Model Name | Discrimination | | Accuracy / Calibration | | Clinical utility | |
| --- | --- | --- | --- | --- | --- | --- |
|  | AUC | F1 | Adjusted Brier Score | Adjusted ECE | NB (overall) | NB (treated) |
| XGBoost-20 | 0.836 (0.835,0.836) | 0.275 (0.274,0.276) | 0.976 (0.976,0.976) | 0.996 (0.996,0.996) | 0.903 (0.903,0.903) | 0.004 (0.0006, 0.0002) |
| XGBoost-23 | **0.837 (0.836,0.837)** | **0.277 (0.276,0.278)** | **0.976 (0.976,0.976)** | **0.995 (0.995,0.995)** | **0.903 (0.903,0.903)** | **0.020 (0.025, 0.015)** |
| XGBoost-25 | 0.837 (0.836,0.837) | 0.278 (0.277,0.280) | 0.976 (0.976,0.976) | 0.995 (0.995,0.995) | 0.903 (0.9026,0.9031) | 0.020 (0.025, 0.015) |
| XGBoost-27 | 0.837 (0.837,0.838) | 0.277 (0.276,0.279) | 0.976 (0.976,0.976) | 0.995 (0.995,0.995) | 0.903 (0.903,0.903) |  |

1. Testing Dataset

| Model Name | Discrimination | | Accuracy / Calibration | | | Clinical utility |
| --- | --- | --- | --- | --- | --- | --- |
|  | AUC | F1 | Adjusted Brier Score | Adjusted ECE | | (Net benefit overall) |
| XGBoost-20 | 0.846 (0.845,0.847) | 0.286 (0.284,0.287) | 0.977 (0.977,0.977) | 0.995 (0.995,0.995) | | 0.907 (0.907,0.908) |
| XGBoost-23 | **0.846 (0.845,0.846)** | **0.288 (0.287,0.290)** | **0.977 (0.9770,0.977)** | | **0.995 (0.995,0.995)** | **0.907 (0.907,0.907)** |
| XGBoost-25 | 0.846 (0.845,0.847) | 0.286 (0.284,0.288) | 0.977 (0.977,0.977) | | 0.995 (0.995,0.995) | 0.907 (0.907,0.907) |

Supplementary Table 4. Shapley importance values of variables used in in the XGBoost-23 model.

| **Variable Name** | **Feature Importance SHaP Value** |
| --- | --- |
| Operation | 0.417 |
| Age | 0.242 |
| CrCl | 0.239 |
| Urgency | 0.233 |
| NYHA | 0.163 |
| CPS | 0.079 |
| PVD | 0.071 |
| Previous Operation | 0.067 |
| Previous MI | 0.055 |
| Cardiac Rhythm | 0.047 |
| Pulmonary Disease | 0.044 |
| First Operator Grade | 0.039 |
| Number of Grafts | 0.037 |
| Diabetes | 0.033 |
| BMI | 0.033 |
| Mitral valve procedure | 0.031 |
| Hospital Code | 0.027 |
| Pre-operative stroke | 0.016 |
| Left ventricular function | 0.015 |
| Previous valve surgery | 0.012 |
| Previous CABG | 0.009 |
| Aortic arch procedure | 0.007 |
| Mechanical support | 0.004 |

CrCl: creatinine clearance. NYHA: New York Heart Association score. CPS: Critical pre-operative state. PVD: Peripheral vascular disease. MI: Myocardial infarction. CABG: Coronary artery bypass graft

Supplementary Figure 1. Variables utilised to derive the model

1. Hypertension

2. Pulmonary Hypertension

3. Left ventricular function

4. Creatinine clearance

5. Acute aortic syndrome

6. Impeller

7. Other Mechanical Support

8. Ventricular assist device

9. Intra-aortic balloon pump

10. Hospital Code

11. Gender

12. Payer Status

13. Canadian chest score

14. New York Heart Association score

15. Previous myocardial infarction

16. Interval myocardial infarction

17. Percutaneous coronary intervention

18. Previous coronary artery bypass grafting

19. Previous valve

20. Previous ascending aortic or arch surgery

21. Previous descending aorta surgery

22. Previous thoracic surgery

23. Diabetes

24. Smoking

25. Pulmonary Disease

26. Pre-operative Stroke

27. Neurological Dysfunction

28. Peripheral vascular disease

29. Preoperative atrial fibrillation

30. Preoperative ventricular fibrillation or tachycardia

31. Preoperative complete heart block or pacing

32. Presence of left main stem disease

33. Pulmonary artery systolic blood pressure

34. Cardiogenic Shock

35. Ventilated Preoperatively

36. Urgency

37. Previous Operation

38. Basal Metabolic Index

39. Mobility

40. First Operator Grade

41. Coronary artery bypass grafting

42. Number of Valves operated upon

43. Aortic valve procedure

44. Preoperative Dialysis

45. Creatinine

46. Age

47. On pump surgery

48. Critical pre-operative state

49. Endocarditis

50. Recent myocardial infarction

51. Post-Infarct ventricular septal defect

52. Mitral valve procedure

53. Tricuspid valve procedure

54. Pulmonary valve procedure

55. Days Between left heart catheterisation and the Operation

56. Extent of coronary artery disease

57. Nitrates

58. Inotropes

59. Weight

60. Number of Grafts Inserted

61. Aortic root procedure

62. Ascending aorta procedure

63. Aortic arch procedure

64. Descending aorta procedure

65. Abdominal aorta procedure

66. Median Sternotomy

67. Partial Sternotomy

68. Thoracotomy

69. Mini-Thoracotomy

70. Indices of multiple deprivation centile

71. Year of the operation


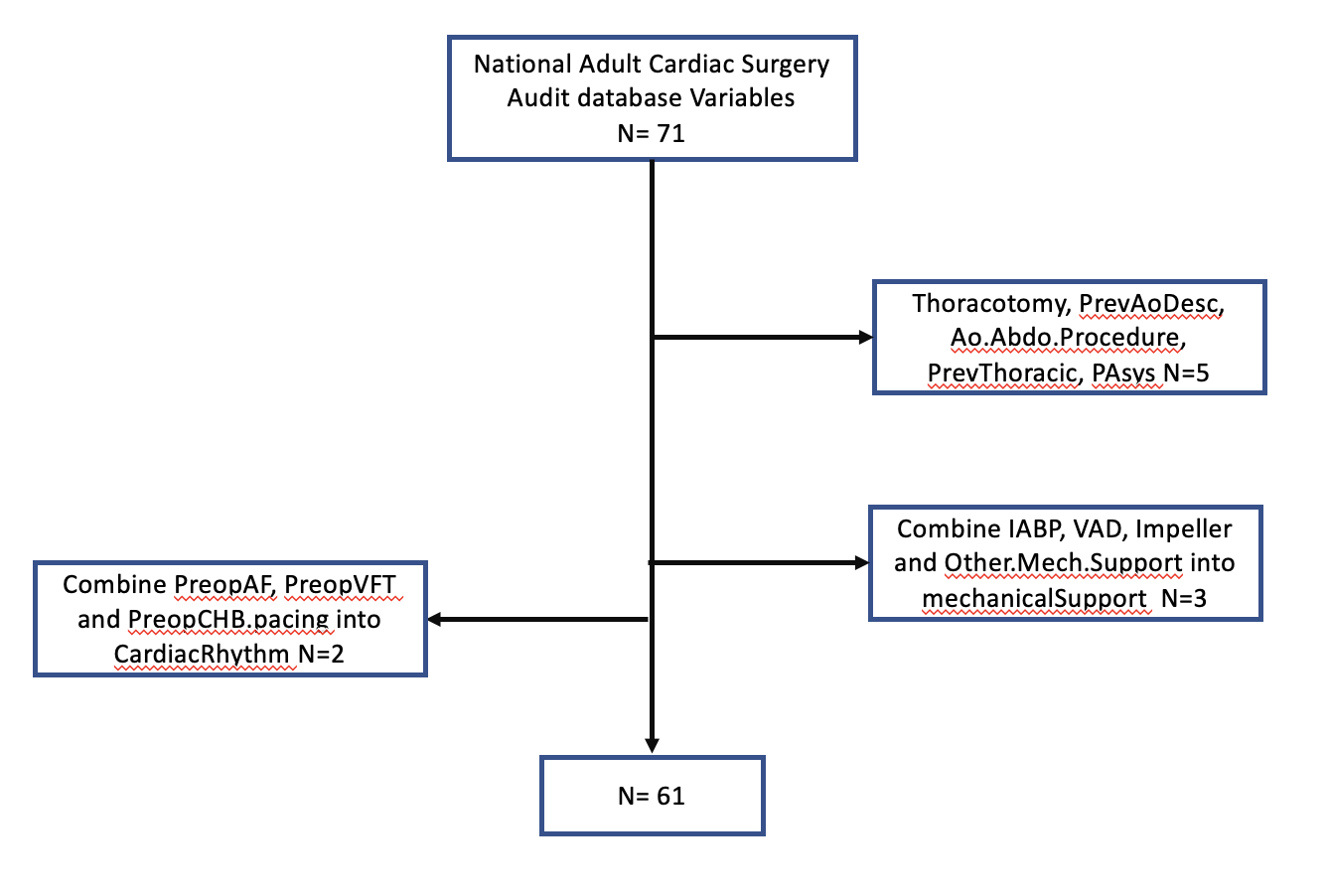


Supplementary Figure 4. Changes in AUC with differing number of variables when developing the XGBoost model with selective backward floating selection of variables.


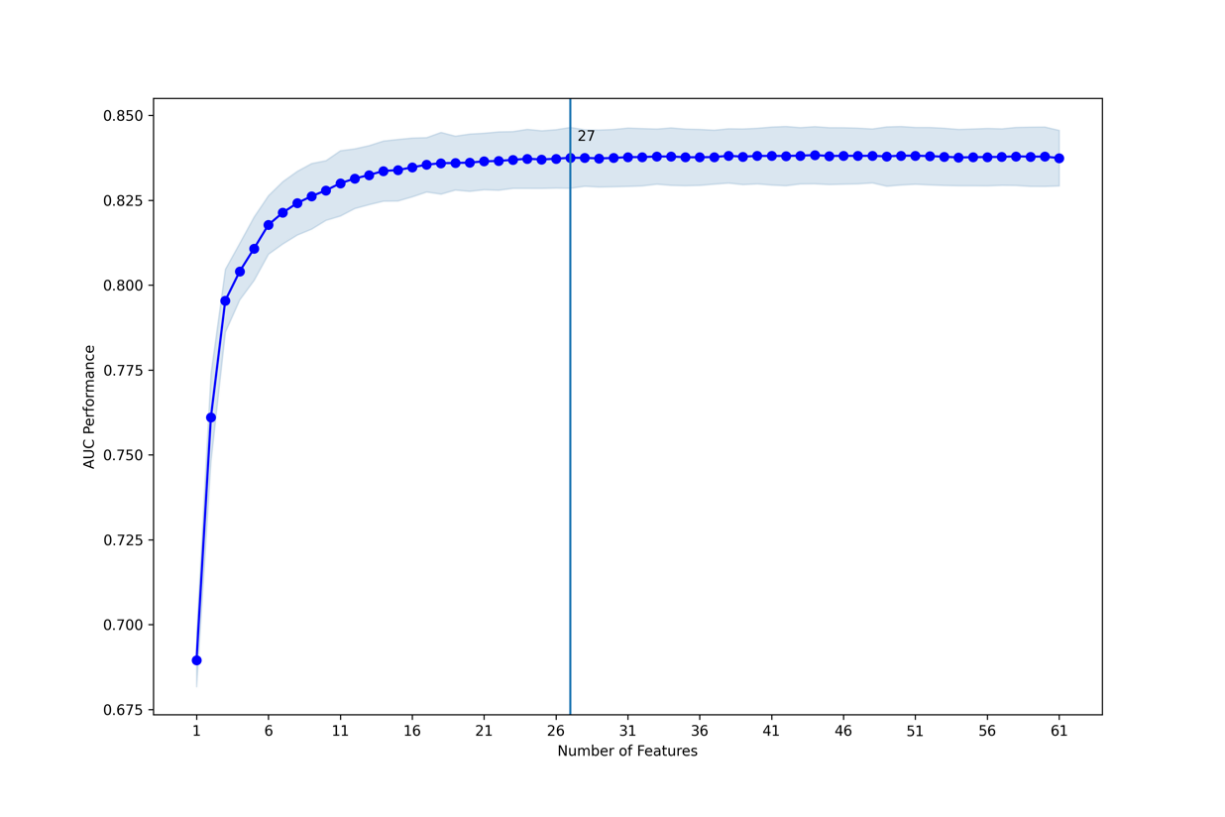


Supplementary Figure 5. Number of mortalities in each decile of risk, as defined by XGBoost-23, in the test group.


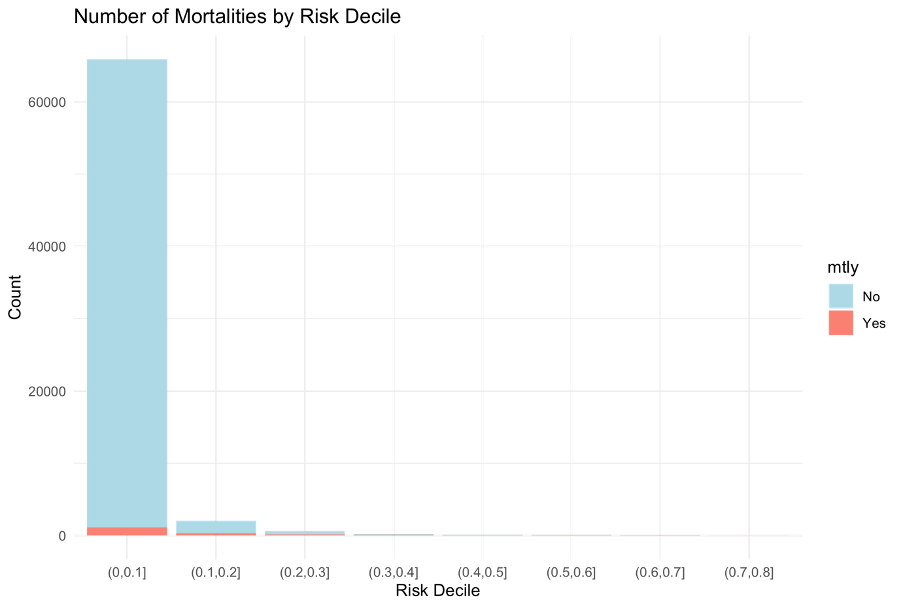

Supplement: ezae368_Supplementary_Data [file ezae368_supplementary_data.docx]
